# Supplementary material for: Low-pass sequencing plus imputation using avidity sequencing displays comparable imputation accuracy to sequencing by synthesis while reducing duplicates
Source: G3 (Bethesda). 2023 Dec 1;14(2):jkad276. doi: 10.1093/g3journal/jkad276 (PMC10849336; doi:10.1093/g3journal/jkad276)
Supplement: jkad276_Supplementary_Data [file jkad276_supplementary_data.pdf]

Supplementary Materials

Jeremiah H. Li, Karrah Findley, Joseph K. Pickrell, Kelly Blease, Junhua Zhao, Semyon Kruglyak

Gencove Inc.

November 27, 2023

1 Figures

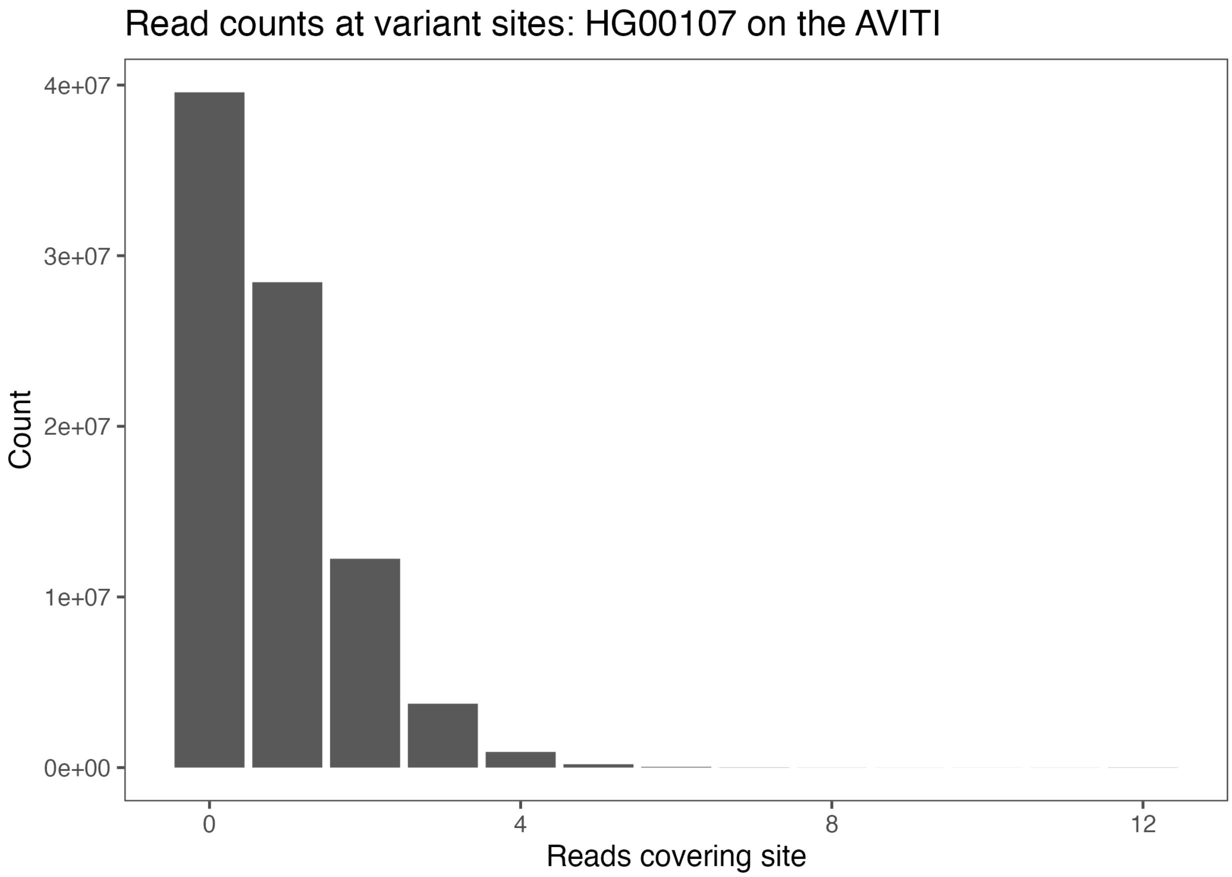

Supplementary Figure 1: Distribution of observed coverage at sites within the reference panel for HG00107 on the AVITI system. Note that the x-axis is truncated at 12 reads as this is the maximum number of reads considered for impute by `loimpute`.

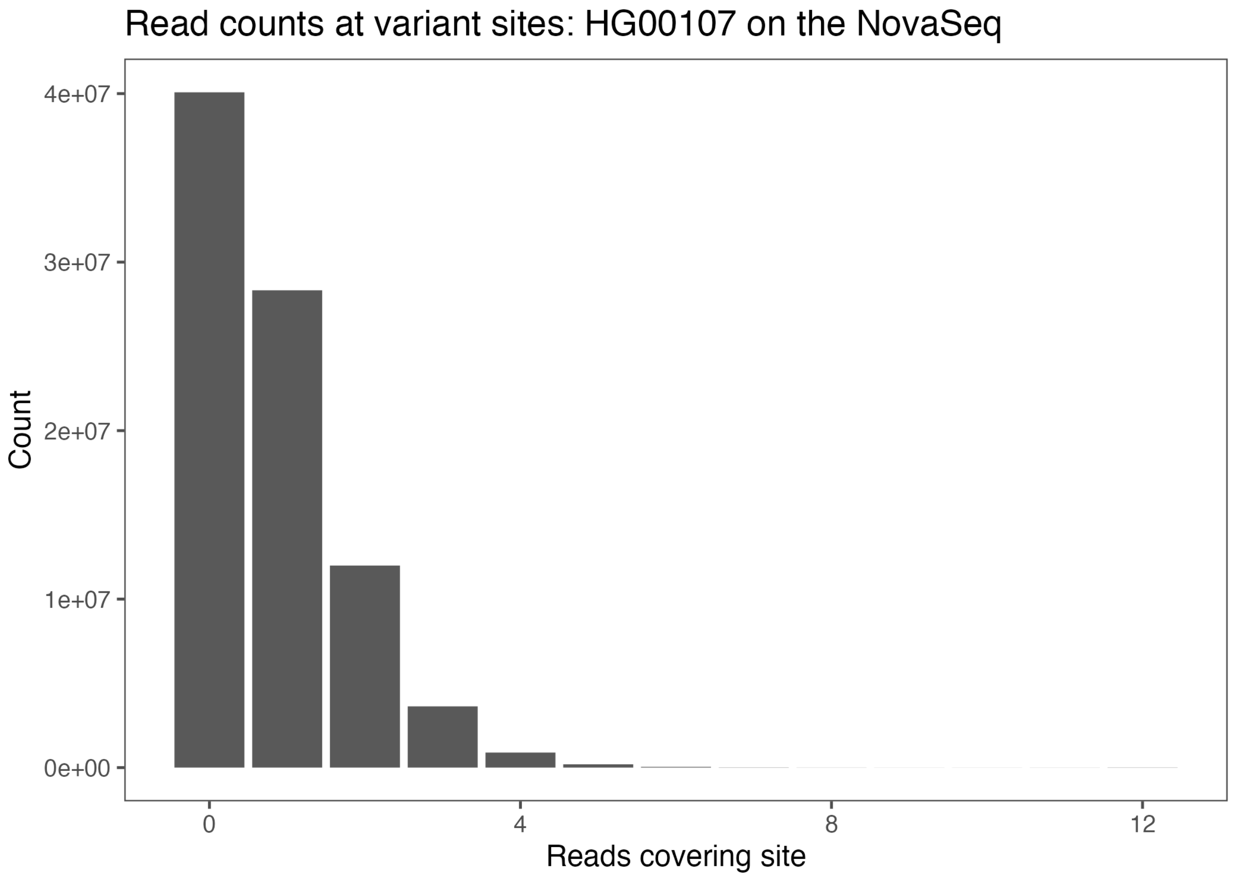

Supplementary Figure 2: Distribution of observed coverage at sites within the reference panel for HG00107 on the NovaSeq. Note that the x-axis is truncated at 12 reads as this is the maximum number of reads considered for impute by `loimpute`.

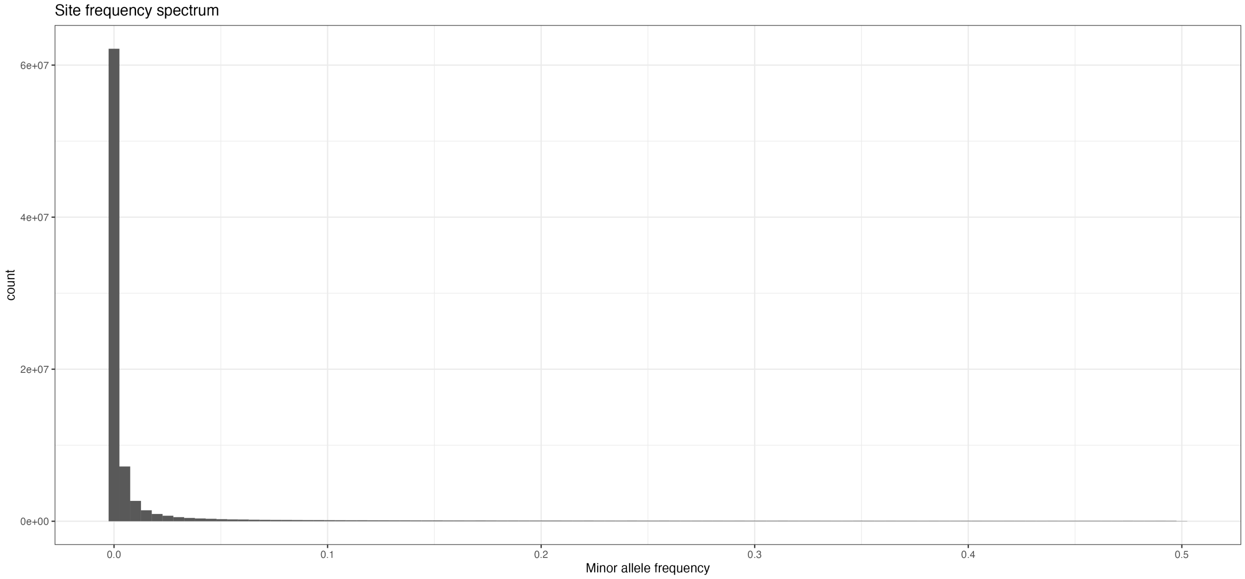

Supplementary Figure 3: Site frequency spectrum of all variants within the 1KGP3 imputation panel.
